# *In Vitro* Study of a Stentless Aortic Bioprosthesis Made of Bacterial Cellulose

**DOI:** 10.1007/s13239-020-00500-z

**Published:** 2020-11-17

**Authors:** Kinga Dawidowska, Piotr Siondalski, Magdalena Kołaczkowska

**Affiliations:** 1 Medical Engineering Division, Maritime Advanced Research Centre, Szczecińska 65, 80-392 Gdańsk, Poland; 2 Cardiac and Vascular Surgery Department, Medical University of Gdańsk, Dębinki 7, 80-211 Gdańsk, Poland

**Keywords:** Human aortic bioprosthesis, HAB, Aortic valve prosthesis, Aortic bioprosthesis, Bacterial cellulose, Nanocelulose

## Abstract

**Purpose:**

The paper present findings from an *in vitro* experimental study of a stentless human aortic bioprosthesis (HAB) made of bacterial cellulose (BC). Three variants of the basic model were designed and tested to identify the valve prosthesis with the best performance parameters. The modified models were made of BC, and the basic model of pericardium.

**Methods:**

Each model (named *V*_1_, *V*_2_ and *V*_3_) was implanted into a 90 mm porcine aorta. Effective Orifice Area (EOA), rapid valve opening time (RVOT) and rapid valve closing time (RVCT) were determined. The flow resistance of each bioprosthesis model during the simulated heart systole, i.e. for the mean differential pressure (Δ*P*) at the time of full valve opening was measured. All experimental specimens were exposed to a mean blood pressure (MBP) of 90.5 ± 2.3 mmHg.

**Results:**

The *V*_3_ model demonstrated the best performance. The index defining the maximum opening of the bioprosthesis during systole for models *V*_1_, *V*_2_ and *V*_3_ was 2.67 ± 0.59, 2.04 ± 0.23 and 2.85 ± 0.59 cm^2^, respectively. The mean flow rate through the *V*_3_ valve was 5.7 ± 1, 6.9 ± 0.7 and 8.9 ± 1.4 l/min for stroke volume (SV) of 65, 90 and 110 mL, respectively. The phase of immediate opening and closure for models *V*_1_, *V*_2_ and *V*_3_ was 8, 7 and 5% of the cycle duration, respectively. The mean flow resistance of the models was: 4.07 ± 2.1, 4.28 ± 2.51 and 5.6 ± 2.32 mmHg.

**Conclusions:**

The *V*_3_ model of the aortic valve prosthesis is the most effective. *In vivo* tests using BC as a structural material for this model are recommended. The response time of the *V*_3_ model to changed work conditions is comparable to that of a healthy human heart. The model functions as an aortic valve prosthesis in *in vitro* conditions.

## Introduction

The rate of aortic valve replacement (AVR) surgeries is growing each year. In the United States between 1999 and 2011 this rate increased on average by 1.6%, and the number of surgical AVRs for elderly patients was also on the rise.^37^ Moreover, during this 13-year-long observation, the number of mechanical prosthetic implants decreased in the US by 28.6% in favour of bioprosthesis implants,^37^ and currently 80% of valve prosthesis implants worldwide are tissue valves.^5^

Bioprostheses are very prone to structural degeneration, and for this reason are recommended to elderly patients, while younger patients need reoperation after some time. The first signs of structural degeneration in porcine aortic valves are observed usually ca. 8 years post-implantation. In bovine pericardial valves degeneration occurs 4 years later,^16^ but this is still not a sufficiently long reoperation-free period for younger patients. That is why many scientists are working on a substitute for a heart valve that would eliminate the need for reoperation.

Polymers are chemical substances that are particularly noteworthy in the above context. Some literature sources emphasize their individual features such as: biocompatibility^6^ and anti-thrombogenicity.^8^ Trileaflet valve prostheses made of silicone were designed in the 1960s,^13^, ^24^, ^31^, ^36^ and first used in clinical trials between 1960 and 1962.^29^, ^30^ In the 1980s studies on silicone prostheses were abandoned for good, mainly because of the very limited valve durability.^7^, ^30^ Interest in polytetrafluoroethylene (PTFE) declined equally fast,^25^ since this material was found to be too rigid and prone to calcification. In the late 1950s the first animal studies were carried out on polyurethane (PUR) mitral and aortic valve prostheses.^1^ The high resistance of this polymer to hydrolysis strongly encouraged further research. In the 1970s trileaflet valve prostheses made of two types of PUR, Lycra Spandex (DuPont) and Biomer (Ethicon), were designed. However, a slight regurgitation was found for the Lycra Spandex prosthesis,^38^ and a less than one year animal study revealed thrombus formation and, similarly to the Biomer valve, the formation of calcium deposits.^15^ Over the years, new heart valve prostheses were made of polyether urethanes (PEUs), and many different types of PUR: polyether urethane ureas (PEUUs), polycarbonate urethane (PCUs), polycarbonate urethane ureas (PCUUs), *etc*.^3^ Scientists also focused on other polymers that combine the properties of silicones and polyurethane materials, i.e. poly(styrene-b-isobutylene-b-styrene), SIBS, characterised by acceptable haemocompatibility, lack of platelet activation in the vascular system, and resistance to calcification.^26^

BActerial SYnthesized Cellulose (BASYC®) is a cellulose-based polymer that found applications mainly in the medical industry. It was successfully tested as a structural material of blood vessels in rats.^17^ Bacterial cellulose (BC) has become the subject of many studies, including those carried out by researchers from London, who have developed a BC nanocomposite with polyvinyl alcohol (PVA) for biomedical use. Depending on the components relative concentration, the behaviour of this composite material during axial tensile test is comparable with the material properties of the porcine aorta and native porcine aortic valve leaflets.^23^

This article presents findings from hydrodynamic tests of an aortic valve prosthesis, further referred to as the HAB (Human Aortic Bioprosthesis), made of pure bacterial cellulose (BC). Currently, there are no reports of the ongoing research on heart valve prostheses made of BC. BC is a natural polymer synthesized by bacteria from low-molecular weight sugars and alcohols. Some bacteria, such as aerobic strain *Gluconacetobacter xylinus* E_25_, synthesize cellulose under natural conditions to protect cells against excessive exposure to light and drying, or to prevent loss of nutrients and oxygen. There are three methods of BC production: static culture, agitating culture, and the airlift reactor.^21^ Three-dimensional structure of BC is based on a scaffold of nanofibrils with a diameter not greater than 100 nm, and therefore it is classified as a nanomaterial. Due to its irregular structure, BC has anisotropic properties, but it is possible to manufacture a cellulose material with fibres arranged in a specific orientation. In contact with water, BC transforms into a hydrogel, and depending on water content, which can reach up to 99%, its mechanical properties change. BC is haemocompatible, which means that it does not induce plasma coagulation, so when in contact with blood it induces destruction of fewer than 2% of blood cells.^27^ BC can be sterilized using standard methods, which eliminates the need for a cross-linking mechanism using glutaraldehyde, contributing to calcification of valve prostheses made of animal tissues. BC is also very easy to suture.

The concept of the aortic valve prosthesis, called HAB, was developed in 2006–2007 at the Medical University of Gdańsk in cooperation with the Ship Design and Research Centre S.A. One of the construction assumption was HAB to be an autologous prosthesis—made of a patient’s pericardium and adjusted to individual needs based on data from imaging studies. The aim of the researchers who coined the concept was to develop a reliable and inexpensive aortic valve prosthesis that could be fabricated following an algorithm to match the needs of individual patients. A characteristic feature of the HAB model, which distinguishes it from the currently used valves, are T-shaped folds designed to reduce the maximum local strains in the aortic wall and to increase the functionality of the prosthesis.^32^*In vitro* pilot studies of the HAB prosthesis, implanted inside a blood vessel dissected from an animal, were carried out using a bovine pericardium as the structural material. The first experiments confirmed the functionality of the HAB prosthesis and the correctness of the design concept.

The aim of the research work was to conduct an experimental *in vitro* verification of the possibility of using bacterial cellulose (BC) as a material for the HAB prosthesis and to indicate the prosthesis model characterized by the highest efficiency of work based on selected indicators. Effective Orifice Area (EOA), rapid valve opening time (RVOT) and rapid valve closing time (RVCT) to evaluate the effectiveness of each model were determined.

## Materials and Methods

### Research Model

This paper presents the results of an *in vitro* study of an HAB aortic valve prosthesis made of BC. BC is a biomaterial made of bacterial cellulose obtained from the agitating culture of *Gluconacetobacter xylinus*. The suspension of starter cells—the inoculum—is applied onto a sterilized production medium and pre-incubated, and then a static culture is carried out in the horizontal bioreactors, during which a cellulose matrix is formed at the liquid–air interface.^19^ The resulting biofilms are purified, soaked in deionized water and then squeezed. Bacterial cellulose produced with this method is characterized by a high content of cellulose, high adhesiveness, flexibility and homogeneity. BC membranes used in this study were manufactured by Bowil Biotech sp. z o.o. (Poland) based on the culture of the bacterial strain Gluconacetobacter xylinus E_25_. Assessment of the usefulness of bacterial cellulose as a new biological implant of the circulatory system was assessed based on the properties of this material.^18^ Resistance to the stretching of BC used as a constructed material of HAB is approx. 22 MPa where resistance to the stretching of pericardium is 12 MPa. The structural resistance of BC is higher than structural resistance of the pig’s aorta. The test of biodegradation properties involving incubation in simulated body fluids for 6 months proved lack of significantly changes—merely an increase in porosity. BC is characterized by low adhesion and thrombogenicity.

**Figure 1:**
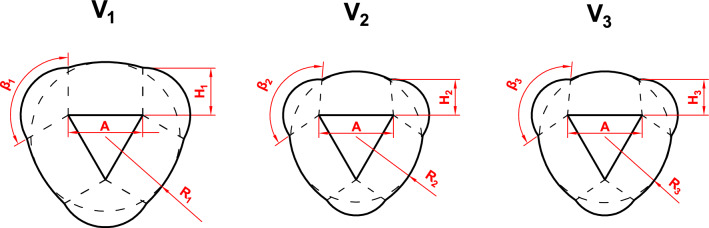
Geometry of a pattern for the fabrication of HABs; *A*—length of the leaflet base, *H*—the leaflet height at the commissure point, *R*—curvature radius of the leaflet free edge, *β*—the angle between the sides of leaflets which are forming the valve fold.

The use of BC instead of the bovine pericardium as the structural material for the HAB caused negative limitations in the functionality of the prosthesis, and hence forced a number of modifications as to the geometrical features and properties of the material. We carried out 12 measurements for the three different geometric models of the HAB, i.e. *V*_1_, *V*_2_ and *V*_3_, for different nominal diameters (20, 22 and 24 mm). A pattern was cut out from BC sheets according to the designed geometry (Fig. 1), then sutures were applied in a way that allowed the formation of folds. Each of the tested prostheses was placed inside an aorta dissected from a pig. Models differed in terms of the height (*H*) and the length of the free leaflet margin of the valve prosthesis. The relationships between the dimensions of the tested models are presented in the Table 1. All models were made of BC cultured under the same conditions. The thickness of the leaflets of all tested models was about 0.1 ± 0.03 mm.

**Table 1:** Parameter relationships for models *V*_1_, *V*_2_ and *V*_3_; *A*—length of the leaflet base, *H*—the leaflet height at the commissure point, *R*—curvature radius of the leaflet free edge, *β*—the angle between the sides of leaflets which are forming the valve fold.

*V*_1_	*V*_2_	*V*_3_
*H*_1_ > *H*_2_	*H*_2_ < *H*_3_	*H*_3_
*R*_1_	*R*_2_ = 0,8 R_1_	*R*_3_ = 1,12 *R*_2_
*β*_2_	*β*_2_ > *β*_1_	*β*_3_ = *β*_2_

Measurements obtained from the tests were used for the assessment of the impact of changes in geometrical parameters on the performance parameters of models, and to identify the most efficient geometric model of the HAB.

### *In Vitro* Studies

**Figure 2:**
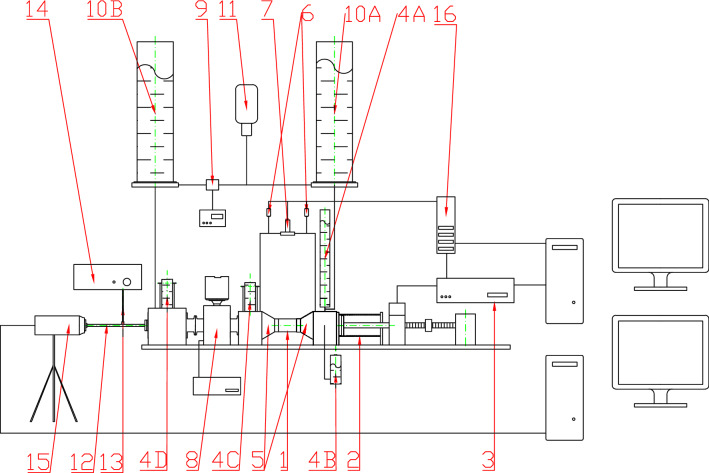
Scheme of the test bench: 1—measuring space; 2—piston pump; 3—piston pump controller 2; 4A–D—control tanks; 5—connectors; 6—absolute pressure transducers; 7—differential pressure transducer; 8—electromagnetic flow meter; 9—ultrasonic flow meter; 10A-B—tanks; 11—gear pump; 12—endoscope; 13—LED light source; 14—optical fibre; 15—camera; 16—CompactRIO system controller.

Studies were conducted on a test bench designed and built at the Maritime Advanced Research Centre in Gdańsk, Poland (Fig. 2). The test bench allowed for the simulation of a flow similar to the blood flow at the outlet of the left ventricle of the human heart. The pulse of the fluid medium and its stroke volume (SV) were modified depending on the settings of the piston pump controller. The tests were carried out *in vitro*—models of prostheses were sewn into fragments of prepared porcine aortas, which were then placed in the measuring area of the test stand. Aortas were collected from deceased animals and were not performed immediately. The tests were not carried out on blood, and the aorta with the valve prosthesis was not immersed in physiological solution.

**Figure 3:**
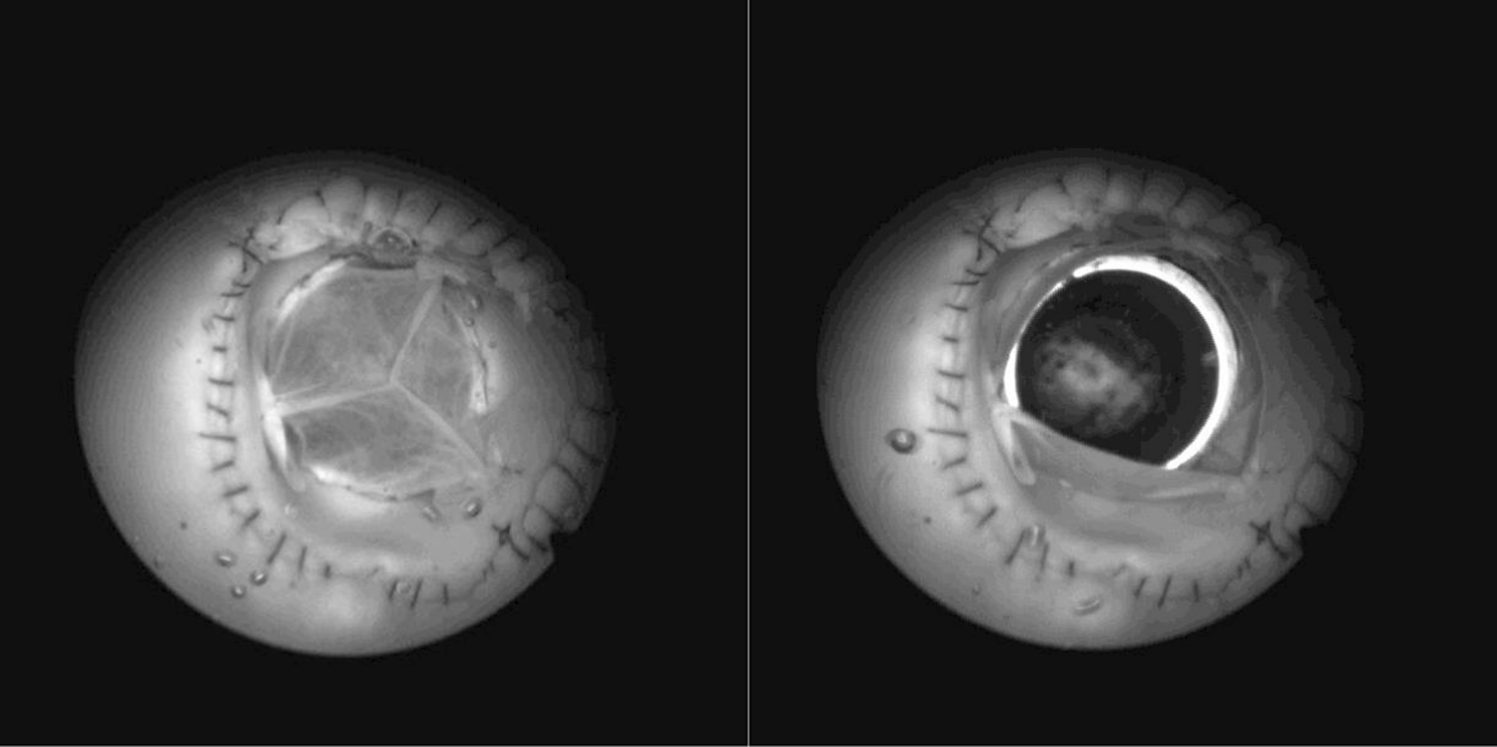
Fully open and closed HAB prosthesis during cardiac working cycle.

*In Vitro* tests were carried out in a working medium, which was water at a temperature of about 20 °C. The flexibility of the flow system corresponding to the flexibility of blood vessels was ensured by the simulator of vascular bed resistance. A video track camera, which is one of the elements of the test bench, allowed for the real-time observation of the performance of the studied prosthesis, and image recording of up to 100,000 frames per second. In Fig. 3 shots from two frames recorded during the research trial of one of the prostheses at the moment when it is fully open and closed are presented. The integrated measuring system registered changes in pressure in the flow channel at the inlet and outlet of the valve prosthesis, and the mean flow rate through the valve. The value of the flow rate through the models resulted from the imposed stroke volume and the set duration of one prosthesis operation cycle. The pressure was the result of these variables and the nominal diameter of the valve prosthesis. The nature of pressure changes could be controlled to a limited extent using the simulator of vascular bed resistance.

During the *in vitro* study we monitored the performance parameters of the three designed models of the aortic valve prosthesis depending on SV (65, 90 and 110 mL), cycle duration (0.6, 0.7 and 0.8 s) and nominal diameter (DN; 20, 22 and 24 mm). The special design of the test bench also allowed for the generation of the mean nominal flow rate for each HAB model under specific work conditions. With these data it was possible to compare different designs of the prosthesis.

Valves performance was assessed based on the designated Rapid Valve Closing time (RVCT) (along with the analysis of changes in pressure values measured upstream and downstream of the prosthesis, on the basis of which the presence of the dicrotic wave of the pulse wave was assessed), the time of its opening—Rapid Valve Opening Time (RVOT), the determined index defining the measure of the prosthesis opening—Effective Orifice Area (EOA), and the amount of flow resistance imposed by the structure at the moment corresponding to the contraction of the heart—the mean value of the differential pressure (ΔPo) during the full opening of the prosthesis.

## Results

The aortic valve prosthesis is deemed functional when its design does not obstruct blood flow during the systole of the left ventricle (does not create an overload to LV), and prevents the backflow of blood to the heart cavities during diastole. The EOA is the standard parameter used for the clinical assessment of aortic stenosis severity and is determined, for example, by Doppler echocardiography. It is the smallest cross-sectional area of the flow behind the valve or valve prosthesis and is the main measure of prosthesis performance tested *in vitro* and *in vivo*. The parameters of the prosthesis orifice area indicated the lowest performance for the *V*_2_ model, for which the EOA was more than 25% smaller than the EOA for the *V*_3_ model (Table 2).

**Table 2:** Comparison of geometrical parameters for different models of aortic valve prosthesis; *Q*—average flow rate, Δ*P*_o_—pressure difference measured at the inlet and outlet of the valve during its full opening, EOA—effective orifice area of the valve, ET—ejection time, RVOT—rapid valve opening time, RVCT—rapid valve closing time.

	*V*_1_	*V*_2_	*V*_3_
*Q* [l/min]	6.51 ± 1.81	7.48 ± 1.76	7.53 ± 1.79
Δ*P*_o_ [kPa]	0.54 ± 0.24	0.57 ± 0.33	0.75 ± 0.31
Δ*P*_o_ [mmHg]	4.07 ± 2.1	4.28 ± 2.51	5.6 ± 2.32
EOA [cm^2^]	2.67 ± 0.59	2.04 ± 0.23	2.85 ± 0.59
ET [ms]	337 ± 42	369 ± 31	321 ± 37
RVOT [ms]	72 ± 29	49 ± 17	51 ± 4
RVCT [ms]	37 ± 7	42 ± 8	38 ± 4

**Figure 4:**
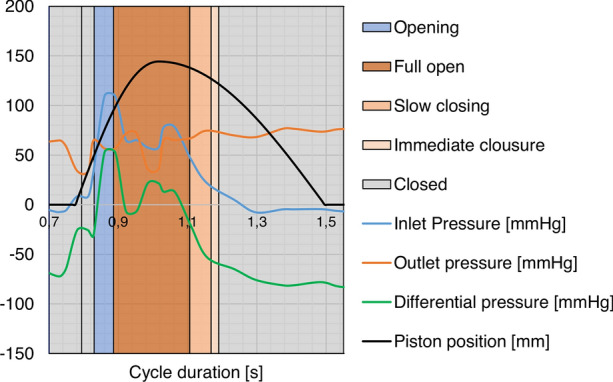
Working cycle of the valve prosthesis and its phases.

Signal registration and frame-by-frame observation of the function of the tested prostheses were used for the comparative analysis of geometric models in terms of the duration of the phases of their work cycle. The following phases of the cycle were specified: closed, opening, fully open, immediate closure and slow closing (Fig. 4).

The performance of a given prosthesis depends on its response time to changing flow parameters through the valve—RVCT and RVOT. Figure 3 presents the mean durations of these phases for all three models of HAB made of BC. Mean opening and closing times for models *V*_1_, *V*_2_ and *V*_3_ were comparable to the relevant times and phases measured for the animal aortic valve.^10^, ^28^ The *V*_3_ model of the aortic valve prosthesis was characterised by the shortest response time to changing flow parameters. Compared to the *V*_1_ model, this time was more than two-fold shorter during the opening phase for lower SV and approx. 10% shorter for higher SV. Extending the opening duration of *V*_1_ could reduce the flow rate through this model. Closing times for all three models were comparable regardless of the simulated hydrodynamic conditions.

The *V*_3_ model showed better performance because it was characterized by the greatest adaptability to changing work conditions. For the *V*_3_ prosthesis, there is a near-linear positive correlation between shorter cycle duration and increased EOA. Changes in the value of this index for the other two tested models are described by different patterns. For the *V*_2_ model a decrease in the index value was observed for shorter cycles. The *V*_1_ model attained the maximum EOA for a 0.7s and shorter cycles. A 9% increase in the EOA between the *V*_3_ and *V*_2_ models, under conditions of the most dynamic simulated flow, was associated with a considerable 20% drop in pressure gradient. This may influence changes in the local distribution of blood flow velocities and thus blood coagulation.

The functional parameters of the aortic valve prosthesis must not overwork the left ventricle. The higher the gradient of pressure measured at the inlet and outlet of the prosthesis during its full opening, the greater the load to the heart. The mean gradient of pressure for each model was not greater than 6 mmHg. The highest gradient of pressure was generated by the *V*_3_ model, but compared to *V*_1_, for which Δ*P*_o_ was the lowest, the mean difference in these parameters was only 1.5 mmHg. The fully open *V*_3_ model of HAB during its operation under various conditions, regardless of the DN, caused a mean 5.6 ± 2.32 mmHg pressure drop in the animal aorta.

## Discussion

The evaluated performance of the tested HAB was comparable to the performance of the aortic valve prostheses used back then.^9^ Three geometrical variants *V*_1_, *V*_2_ and *V*_3_ of aortic valve prosthesis made of BC, were tested *in vitro* in order to compare their performance parameters. In each experiment we simulated the work of the model in an environment similar to that in which the prosthesis is ultimately to be used as a substitute of the aortic valve. Observations were made for models with different nominal dimensions, for the same set parameters of the test bench determining the conditions of medium flow through the tested models of valves. Each valve prosthesis was made of a polymer material manufactured in the process of aerobic bacterial culture and known as bacterial cellulose. Models of HAB tested *in vitro* differ in terms of design parameters which influence the dynamics of flow through these valves. Considering the purpose of these models and the environment in which they are to ultimately work, differences in the parameters of flow through each of them and changes in these parameters during work influence their functionality and performance. The effect of design features on the performance of the valve prosthesis may also determine its service life.

The performance of the *V*_1_ model differs considerably from that of the other two models. When prostheses worked under predefined identical flow conditions characteristic for a young human in a relaxed state, the difference in the performance of the *V*_3_ and *V*_1_ models was up to 19%. When cycle duration was reduced to 0.6 s, the difference between the *V*_3_ and *V*_1_ models was up to 30% for lower stroke volumes.

Shorter work cycle of a prosthesis is associated with its more dynamic work, and thus decreased flexibility of the experimental system. However, this apparently undesirable phenomenon allows for the observation of the prosthesis’ performance under significant differential pressure. Under such conditions the flow rate *Q* generated by the *V*_3_ prosthesis was closest to the nominal flow rate.

There is a linear correlation between the flow rate and stroke volume generated by the pump on the test bench simulating the heart function, which indicates the correct response of the designed HAB prosthesis (regardless of the model) to the set flow parameters. The response time of the *V*_3_ model to changed work conditions is comparable to that of a healthy human heart.^10^, ^20^ On average, this accounts for 5% of the whole cycle duration—the mean RVOT and RVCT are similar. Considering all the tested models of the valve prosthesis, the fastest response to changing flow conditions was found for the *V*_3_ model.

The gradient of pressure measured at the inlet and outlet of the valve prosthesis at the peak flow rate, i.e. when the valve is at the full open phase, is the parameter indicating the flow resistance created by the fabricated prosthesis. The lower the value of this flow resistance, the lower the degree of load on the left ventricle resulting from the operation of the valve prosthesis. In order to assess the performance of valve prosthesis models, the mean Δ*P*_o_ determined based on data from measurements was compared with measurements taken during the monitoring of patients with implanted stentless aortic valve bioprostheses and reported in the literature (Table 3). The mean Δ*P*_o_ for each of the three HAB models was lower than the lowest differential pressure measured over 1-year operation of the Medtronic Freestyle prosthesis. Interestingly, ΔP_o_ measured in patients decreased throughout the post-implantation period.

**Table 3:** Mean gradients of pressure for stentless aortic valve bioprostheses (± standard deviation).

Prosthesis model	Time of measurement	Δ*P*_o_ (kPa)	Δ*P*_o_ (mmHg)	References
Medtronic Freestyle	At discharge	1.33 ± 0.8	10 ± 6.0	11
	3–6 months	0.93 ± 0.67	7 ± 5.0	
	1 year	0.8 ± 0.53	6 ± 4.0	
	2 years	0.8 ± 0.67	6 ± 5.0	
Edwards Prima	1 week	2.93 ± 0.67	22 ± 5.0	4
	6 months	2.19 ± 0.93	16.5 ± 7.0	
	1 year	1.93 ± 0.93	14.5 ± 7.0	
Edwards Prima Plus	discharge	2.39 ± 1.19	18 ± 9.0	34
	1 year	1.59 ± 0.53	12 ± 4.0	
	6 months	1.64 ± 0.53	12.3 ± 4.0	12
	1 year	1.28 ± 0.8	9.6 ± 6.0	
Sorin Pericarbon Freedom	at discharge	1.47 ± 0.23	11 ± 1.7	33
	14 years	1.19 ± 0.32	9 ± 2.4	
	10 years	1.19 ± 0.8	9 ± 6.0	22
Sorin Freedom Solo	At discharge	0.87 ± 0.57	6.5 ± 4.3	14
	1 month	0.87 ± 0.51	6.5 ± 3.8	2
	1 year	0.89 ± 0.55	6.7 ± 4.1	
	1 year	1.05 ± 0.33	7.9 ± 2.5	35
	5 years	1.03 ± 0.48	7.7 ± 3.6	

*V*_1_, *V*_2_ and *V*_3_ models were selected for *in vitro* tests based on previously conducted pilot studies. The authors assessed them as the most optimal design solutions. The conducted *in vitro* studies show that seemingly small changes in the geometry of the HAB aortic valve prosthesis affect its operation. They affect its effectiveness—changing the EOA by up to 28% (*V*_3_ vs. *V*_2_) or the time of opening the prosthesis by less than 30% (*V*_1_ vs. *V*_3_). *V*_3_ model has the functionality of an aortic valve under *in vitro* conditions. Long-term fatigue tests should be carried out to assess the suitability of HAB prosthesis model *V*_3_ made of BNC as a substitute for the aortic valve in the circulatory system. Due to the more frequent minimally invasive procedures and transcatheter implantation of heart valve prostheses, it is recommended to determine the possibility of using this method of implantation for the HAB prosthesis.
